# Mechanism of salidroside in the treatment of endometrial cancer based on network pharmacology and molecular docking

**DOI:** 10.1038/s41598-023-41157-7

**Published:** 2023-08-29

**Authors:** Panpan Yang, Yihong Chai, Min Wei, Yan Ge, Feixue Xu

**Affiliations:** 1 Department of Obstetrics and Gynecology, First Hospital of Lanzhou University, Lanzhou, 730000 Gansu People’s Republic of China; 2 The First Clinical Medical College of Lanzhou University, Lanzhou, 730000 Gansu People’s Republic of China

**Keywords:** Molecular medicine, Cancer, Cancer prevention

## Abstract

Salidroside is a natural product of phenols, which has a wide scape of pharmacological effects, but its pharmacological effects and molecular mechanism on endometrial cancer are not clear. To systematically explore the pharmacological effects and molecular mechanisms of salidroside on endometrial cancer through the method of network pharmacology. The possible target genes of salidroside were obtained through different pharmacological databases and analysis platforms, and then the relevant target genes of endometrial cancer were obtained through the GeneCards website, and the target genes were uniformly converted into standardized gene names with Uniprot. The collected data were then processed to obtain common target genes and further analyzed through the String website to construct a protein–protein interaction (PPI) network, followed by gene ontology (GO) functional annotation and Kyoto Gene and Genome Encyclopedia (KEGG) pathway analysis. We further interpreted the molecular mechanism of salidroside for the treatment of endometrial cancer by constructing a “drug component–target gene–disease” network. Finally, we performed molecular docking to validate the binding conformation between salidroside and the candidate target genes. There were 175 target genes of salidroside after normalization, among which 113 target genes interacted with endometrial cancer. GO analysis indicated that the anti-endometrial cancer effect of salidroside may be strongly related to biological processes such as apoptosis and response to drug. KEGG analysis indicated that its mechanism may be related to pathway in cancer and PI3K-AKT signaling pathway. Molecular docking showed that salidroside had high affinity with five key genes. Based on the novel network pharmacology and molecular docking validation research methods, we have revealed for the first time the potential mechanism of salidroside in the therapy of endometrial cancer.

## Introduction

Endometrial cancer (EC) is a malignant tumor of the endometrial epithelium, which is more common in postmenopausal women and is one of the three major malignant tumors of the female reproductive tract. In recent years, the incidence of EC has been increasing each year, and there is a younger trend^1^. The American Cancer Society estimates that by 2022, there will be 65,950 new cases and 12,550 deaths from EC in the United States, making it the fourth most common cancer and the sixth most common cause of cancer death in women^2^. Currently, operative treatment is the major intervention for EC, and chemotherapy and hormone therapy are the main adjuvant treatments for women with advanced and recurrent disease^3^. However, there are still many controversies surrounding the treatment of endometrial cancer, including the assessment of lymph nodes and the choice of adjuvant therapy^4^. In addition, the treatment options for advanced and recurrent EC are more limited. Given the controversial and limited therapeutic options for EC, there is an urgent demand for development of novel natural medicines targeting biological targets for EC diagnosis, treatment, and prognosis.

In recent years, a wide range of studies have reported that traditional Chinese medicine and its components have significant antitumor effects, including blocking the cell cycle, promoting cell differentiation, inducing apoptosis or autophagy, and reversing chemotherapy resistance^5–10^. Salidroside (SAL) is an active component isolated from the rhizome of *Rhodiola rosea*. More and more studies have shown that SAL not only has the functions of anti-hypoxia, anti-inflammation, anti-aging, and immune enhancement, but also has various pharmacological effects such as immune regulation and anti-cancer^11,12^. Therefore, SAL is a promising anticancer agent. Studies have shown that SAL has demonstrated significant anticancer effects in various cancer cell lines such as ovarian cancer, breast cancer, bladder cancer, colorectal cancer, and leukemia^12–14^. Different concentrations of SAL were used to treat human bladder cancer cell line T24, and the results showed that SAL was able to interfere with a variety of biological behaviors of T24 in vitro culture by mechanisms related to PI3K/AKT and BCL2 signaling pathways^15^. Sun et al. found that SAL acts on a nude mouse transplantation model of breast cancer MCF-7 cells to induce apoptosis by upregulating bax, caspase3 and downregulating bcl-2, p53 expression. It was also found that high dose of SAL could inhibit angiogenesis^16^. Tumor angiogenesis can promote tumor growth, suggesting that anti-angiogenesis may be one of the anti-tumor mechanisms. SAL can induce cell cycle arrest and promote apoptosis after acting on MDA-MB231 and MCF-7 cell lines^17^. Yang et al. showed that SAL reduced the viability of gastric cancer cells, promoted apoptosis, and inhibited proliferation, migration, and invasion by treating gastric cancer cells (SNU-216, MGC-803) with SAL. It also down-regulated p21,bcl-2,MMP2,RhoA,p-ROCK1,Vimentin,CyclinD1,Cleaved-caspase, and further found that SAL inhibited gastric cancer cells by regulating miR-99a/IGF1R and inhibiting MAPK,ERK,PI3K/AKT signaling pathway^18^. At present, the specific mechanism of SAL anti-tumor effect is still unclear, and SAL has not been studied in the treatment of EC.

Network pharmacology is a drug research method developed on the basis of bioinformatics, systems biology, and pharmacology^19^. Adopting a systems biology and bioinformatics approach, the study is based on the “disease–target gene–drug” interaction network from a holistic perspective, and through network analysis, we can systematically and comprehensively observe the intervention and impact of drugs on the disease network and reveal the mechanism of multi-molecular drug synergy in the human body. Network pharmacology can analyze and interpret the mechanism of action of Chinese medicines in a holistic manner, which is conducive to promoting in-depth research and expanding the development of clinical indications of Chinese medicines. Molecular docking is a research method based on an in silico structural theory framework, which simulates the process of docking small molecules into large molecule structures to score their complementary values at the binding site^20^. It is a field of dynamic research and is the most attractive tool in the field of structure-based drug design, lead optimization, biochemical pathways and drug design^21^. Molecular docking technology is now widely used in drug development to identify new compounds of therapeutic interest, predict ligand–target interactions at the molecular level, or characterize their conformational relationships^22^.

**Figure 1:**
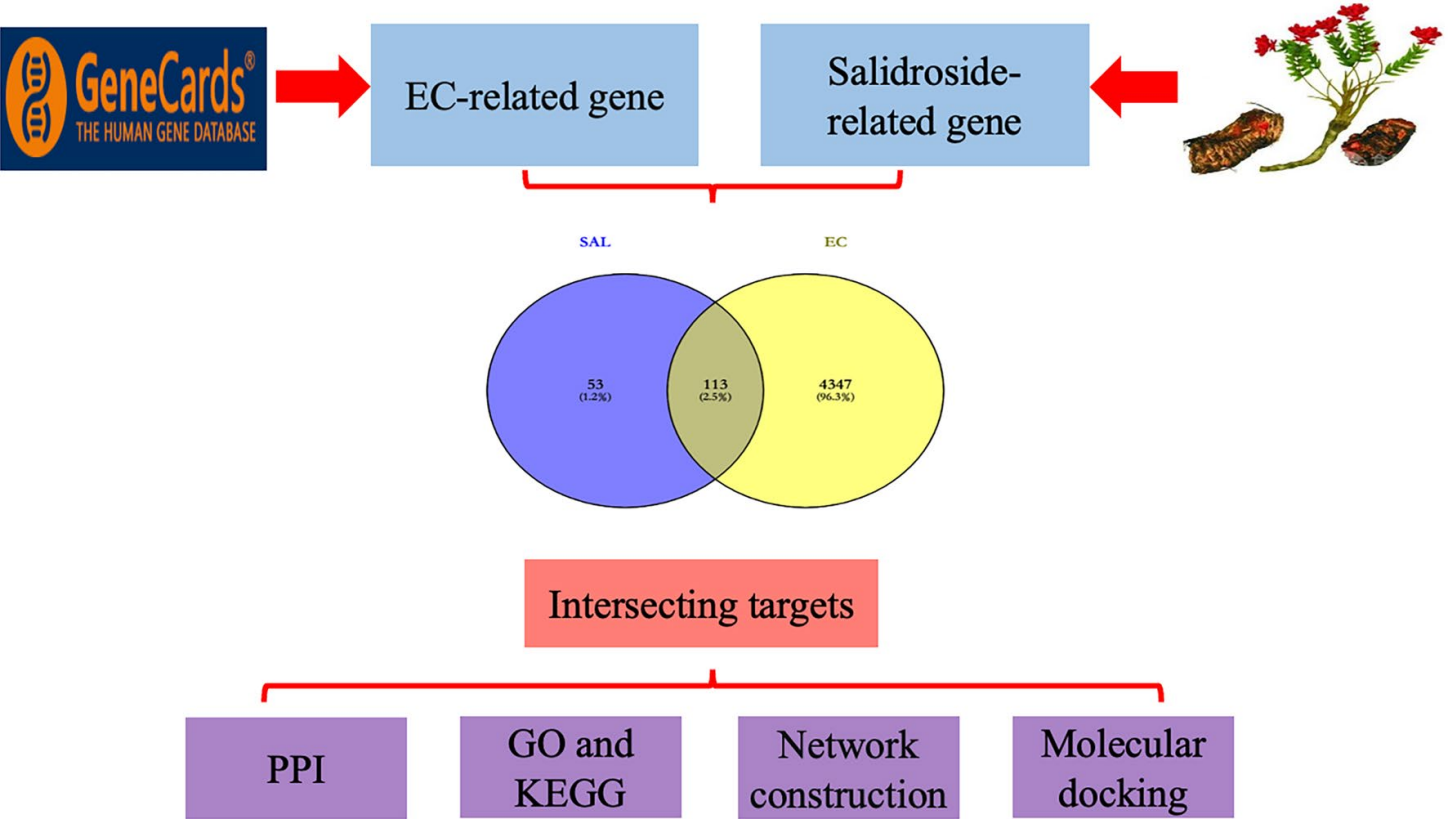
The research flowchart.

Therefore, this study aims to combine network pharmacological analysis with molecular docking to explore the possible pharmacodynamic substances and potential targets of action of SAL for the treatment of EC for the first time, and to provide a theoretical basis for the development of new drugs for the treatment of EC. The overall concept of the research is depicted in Fig. 1.

## Materials and methods

### Collection SAL and EC target genes

The CAS (10338-51-9) of SAL was entered into the TCMSP (TCMSP—Traditional Chinese Medicine Systems Pharmacology Database and Analysis Platform (tcmsp-e.com)) database. The SMILE formula of SAL was imported into the Swiss Target Prediction database (SwissTargetPrediction). The SDF format of SAL was downloaded from the PubChem (PubChem (nih.gov)) database and entered into PhamMapper (PM—Submit Job (lilab-ecust.cn)). The CTDBASE (The Comparative Toxicogenomics Database | CTD (ctdbase.org)) database was searched for SAL-related target genes using the keyword “salidroside”. The relevant target genes of SAL were obtained separately. The target genes related to EC were retrieved in Gene Cards (Account—GeneCards Suite) database with “endometrial cancer” as the keyword. Using Uniprot data, the obtained gene names were corrected to their official names (office symbol). The obtained SAL targets and EC targets were intersected, and the venn diagram of the intersected targets was displayed using the Venny (2.0.1) online tool.

### Construction of target genes protein interaction network

The obtained intersecting genes were imported into String (STRING: functional protein association networks (string-db.org)) database to construct PPI network, and selected species: homo sapiens with a composite score greater than 0.4 as a qualifying condition to obtain protein interaction information. The PPI network was then visualized and analyzed by importing the protein interaction information into Cytoscape 3.8.2 software to obtain key genes. The topological parameters of the network were calculated by the "Cyto NCA" module in cytoscape software, and the degree of connectivity (Degree) was used as a quantitative reference for the importance of nodes, and then the important targets were selected. A hub genes diagram is drawn using the Cyto Hubba plugin in cytoscape software.

### GO and KEGG analysis

Gene Ontology (GO) functional analysis was performed using the David ((DAVID Functional Annotation Bioinformatics Microarray Analysis (ncifcrf.gov))) database^23,24^, including biological process (BP), molecular function (MF), and cellular component (CC) analysis, with *P* < 0.05 was used as the screening condition, and the bubble diagram was drawn by taking the top 20 entries of different biological processes according to the *P* value. Meanwhile, the Kyoto Encyclopedia of Genes and Genomes (KEGG) pathway enrichment analysis was performed, and *P* < 0.05 was used as a significant enrichment screening condition to obtain the main pathways of SAL treatment EC^25^. The top 20 signaling pathways were selected to draw bubble maps according to *P* values. GO and KEGG was plotted by https://www.bioinformatics.com.cn, an online platform for data analysis and visualization.

### Construction of “drug–target–disease” network

The “drug–target–disease” network was built with Cytoscape 3.8.2. We imported common target genes into Cytoscape 3.8.2 to build a multi-level network. the software core of Cytoscape provides the basic functions of laying out and querying the network, visualizing, and integrating the network with expression profiles, phenotypes, and other molecular states. The CTD network map provides a more visual and accurate representation of the relevant targets for drug interventions in diseases.

### Correlation analysis between genes and phenotypes

We use VarElect module in GeneCards to analyze the correlation between key genes and diseases.

### Molecular docking

The 2D structure of SAL (MOL003341) was downloaded with PubChem website, and the compound structure was converted to 3D structure by software Chem3DUltra 14.0. The crystal structures of top 5 core proteins (AKT1 (PDB ID: 1UNQ), HIF1A (PDB ID: 4H6J), caspase 3 (PDB ID: 2DKO), EGFR (PDB ID: 8A27), MMP9 (PDB ID: 6ESM)) were downloaded from Protein Data Bank (RCSB PDB: Homepage). These five target protein receptors were then treated with Pymol software, such as dehydration and removal of organic matter. The target protein receptor molecules were hydrogenated, and charge calculated by AutoDock Tools 1.5.7, and the compounds and target protein receptors were converted to “pdbqt” files with appropriate box center and box point parameters. Finally, Vina 1.5.7 was run to evaluate molecular docking. The best docking results for each core target protein and compound binding are visualized by Pymol and Ligplot software. The lower the score, the higher the affinity.

## Results

### Acquisition target genes

**Figure 2:**
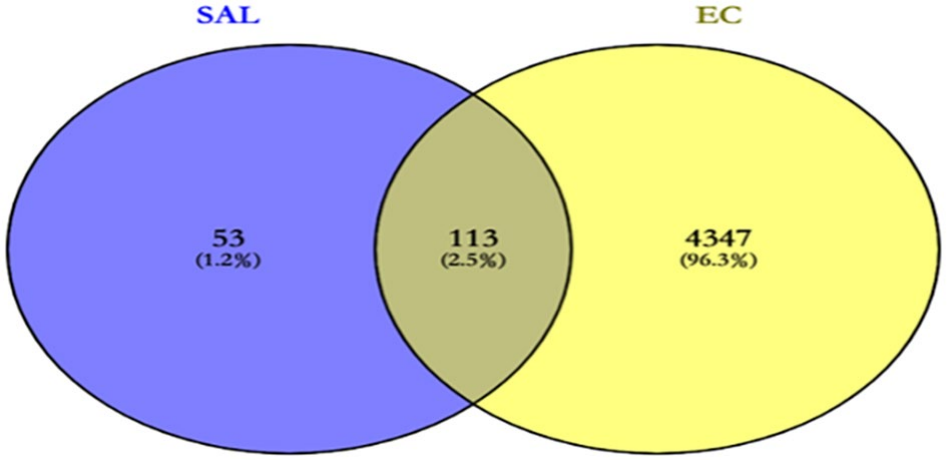
Intersecting targets.

175 SAL-related target genes and 4460 EC-related target genes were obtained through different databases, respectively (Supplementary Data 1). Finally, 113 targets in SAL were obtained by plotting Venn diagrams as potential targets for treating EC (Fig. 2).

### Construction of protein interaction network

**Figure 3:**
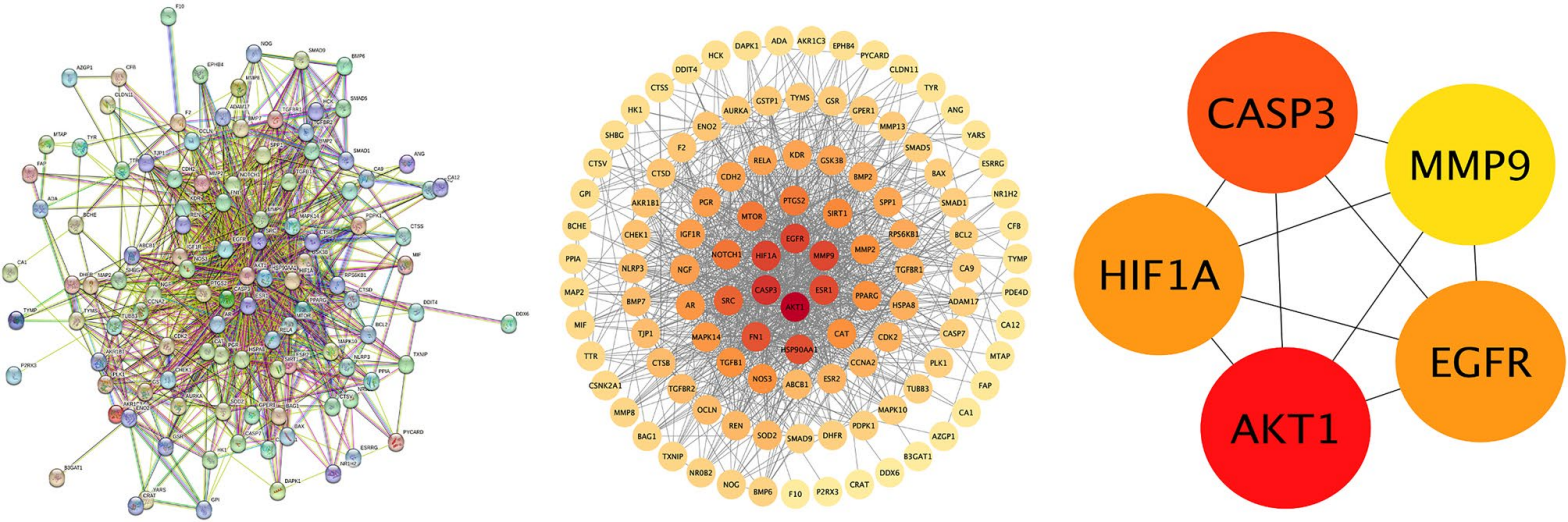
PPI network and core target genes.

The protein interaction network of 113 targets was constructed by Sting combined with Cytoscape software (Fig. 3). Topological analysis of the PPI network based on cyto-NAC function was performed to obtain the Degree values of all target genes, and the top five target genes in the Degree ranking (AKT1, CASP3, EGFR, HIF1A, and MMP9) were taken as the key acting targets for SAL treatment of EC.

### GO and KEGG enrichment analysis

**Figure 4:**
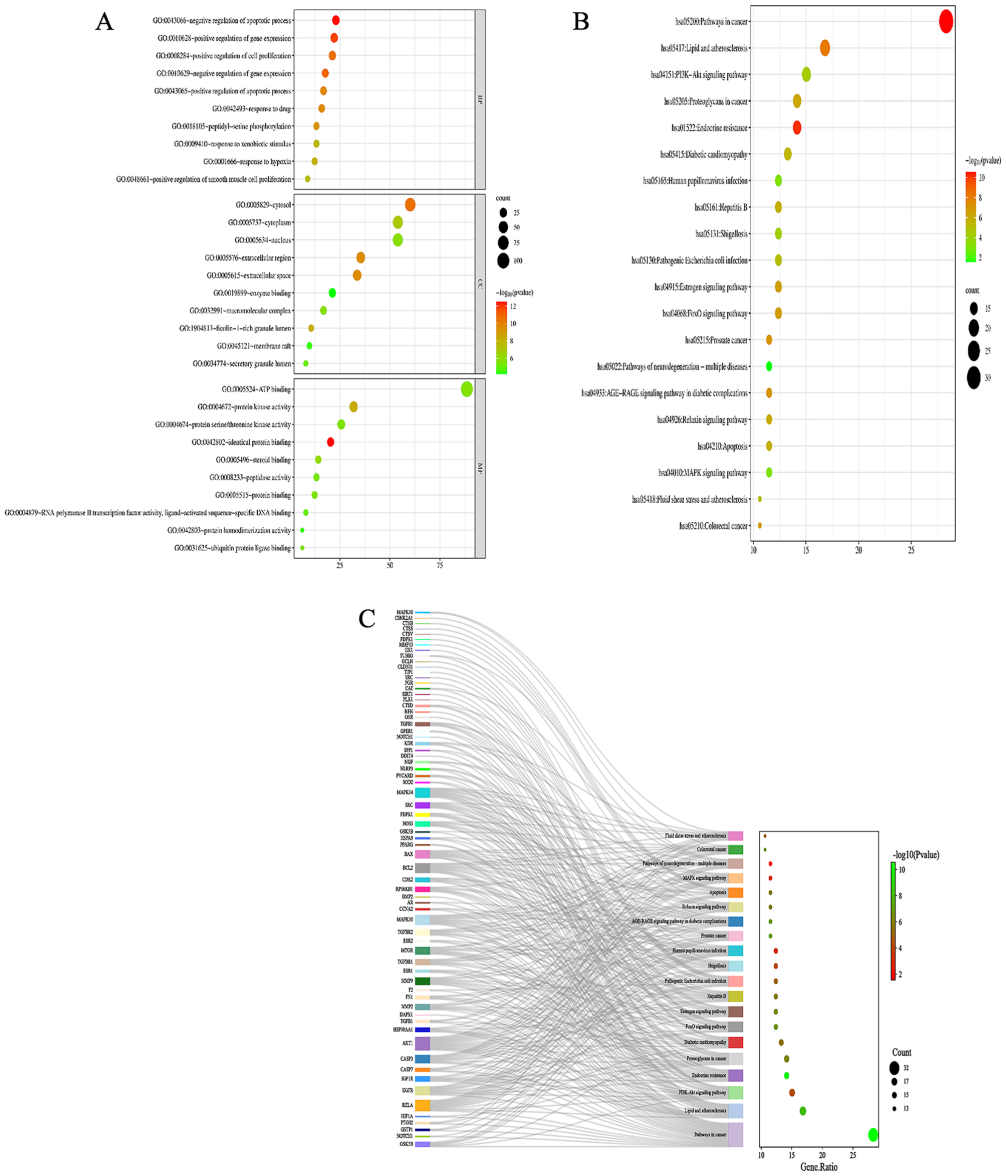
GO and KEGG analysis.

GO and KEGG analysis were performed on 113 intersection target genes through the David website. A total of 531 BP involved in SAL treatment of EC, mainly including: negative regulation of apoptotic process, positive regulation of gene expression, response to drug, etc. AKT1, CASP3, EGFR and HIF1A are closely related to tumor cell apoptosis, and HIF1A and MMP9 are closely related to tumor metastasis; A total of 71 CC, mainly including: cytosol, cytoplasm, extracellular exosome, etc. AKT and HIF are in the cytoplasm, EGFR in the cell membrane, which is closely related to cell growth and differentiation, CASP3 in the cytoplasm and MMP9 in the extracellular fluid. A total of 93 MF, mainly including: enzyme binding, protein kinase activity, protein binding, etc. (Fig. 4).

**Figure 5:**
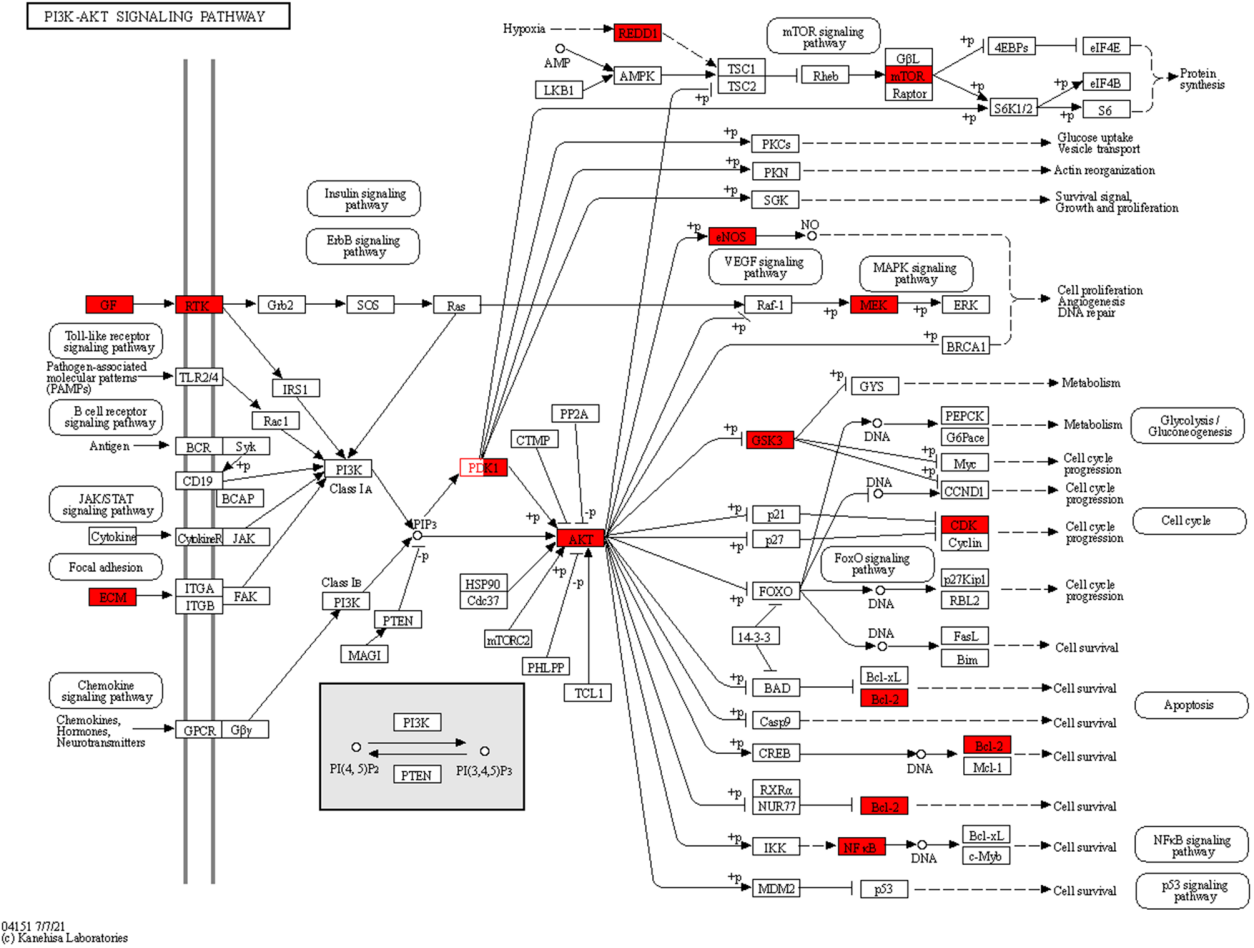
Target genes in the KEGG signaling pathway (PI3K/AKT signal pathway).

Using *P* < 0.05 as the screening condition, 144 enriched signaling pathways were screened (Fig. 4). The signaling pathways in which SAL exerted anti-EC were found to include the pathway in cancer, PI3K-AKT signal pathway, apoptosis, MAPK signal pathway, FOX signal pathway etc. AKT1, CASP3, EGFR, HIF1A, and MMP9 were significantly enriched in PI3K/AKT signaling pathway, pathway in cancer, apoptosis, MAPK signaling pathway, and FOX signaling pathway. PI3K/AKT signaling pathway is closely related to pathway in cancer, apoptosis, MAPK signaling pathway, and FOX signaling pathway, and is involved in malignant biological behaviors such as tumor proliferation, apoptosis, and metastasis, suggesting that SAL may play an anti-EC role through PI3K/AKT signaling pathway^26,27^ (Fig. 5).

### Construction of drug–target–disease network

**Figure 6:**
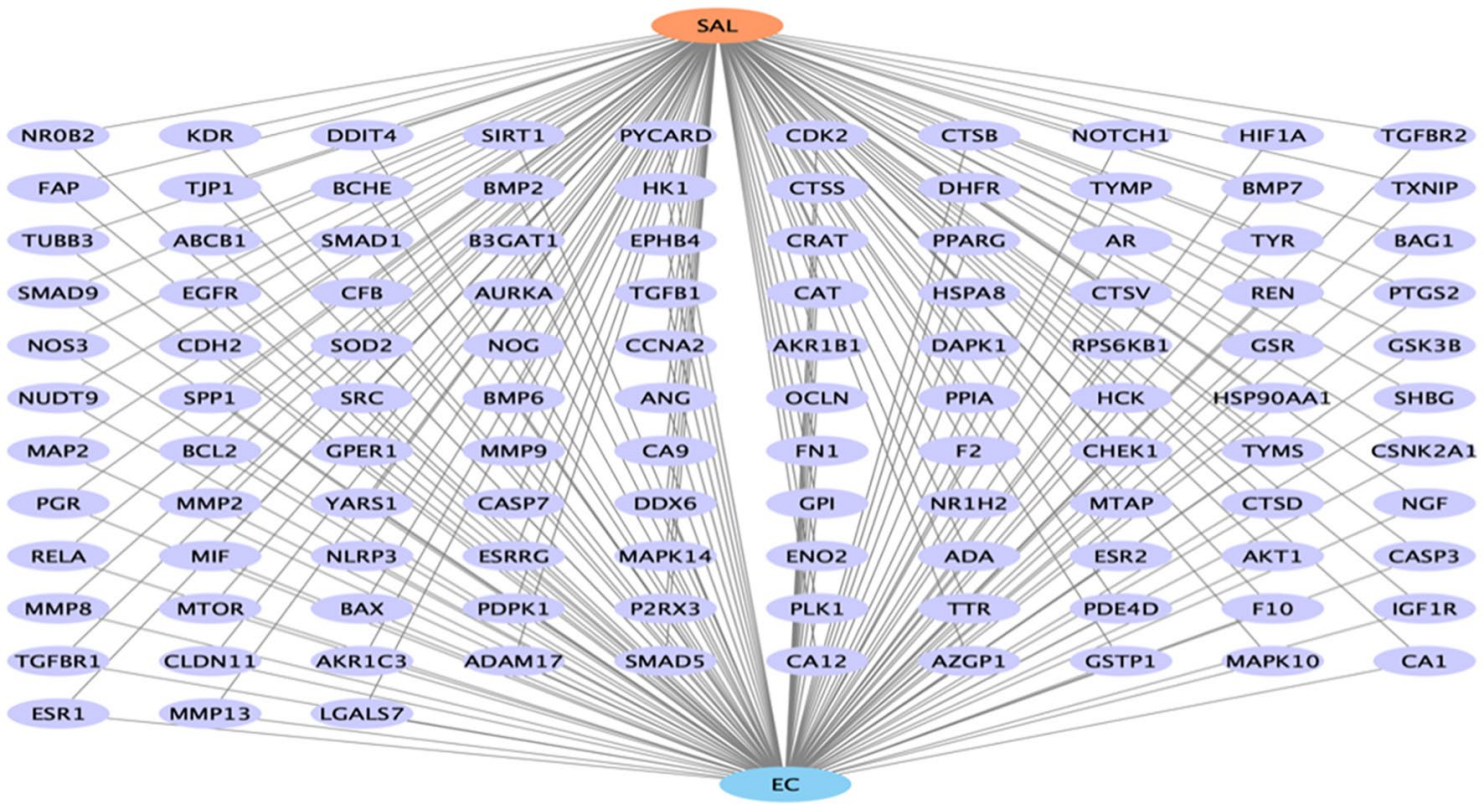
The “drug–target–EC” network.

To further explore the molecular mechanism of SAL for EC treatment, we constructed a “SAL–target gene–EC” network (Fig. 6). The purple nodes represent common target genes, the orange nodes represent SAL, the blue nodes represent EC, and the edges represent interactions (Fig. 6). The construction of the “drug–target–disease” network can show the relevant targets of SAL for EC treatment more visually.

### Correlation between gene and phenotype

To further evaluate the correlation between key target genes and EC, we conducted a correlation analysis between genes and phenotype. We evaluated the scores of these five central targets using the VarElect module in GeneCards, and the score indicates the strength of the connection between the target and EC. As shown in Table 1, EGFR, AKT1, MMP9, CASP3 and HIF1A genes were all directly associated with EC, and all scored high.

**Table 1:** Correlation of the core genes with the EC phenotype.

Symbol	Description	− LOG10(P)	Score
EGFR	Epidermal growth factor receptor	3.43	136.42
AKT1	AKT serine/threonine kinase 1	3.22	102.60
HIF1A	Hypoxia inducible factor 1 subunit alpha	2.34	41.00
MMP9	Matrix metallopeptidase 9	2.31	40.04
CASP3	Caspase 3	2.24	36.61

### Molecular docking verification of key targets

**Figure 7:**
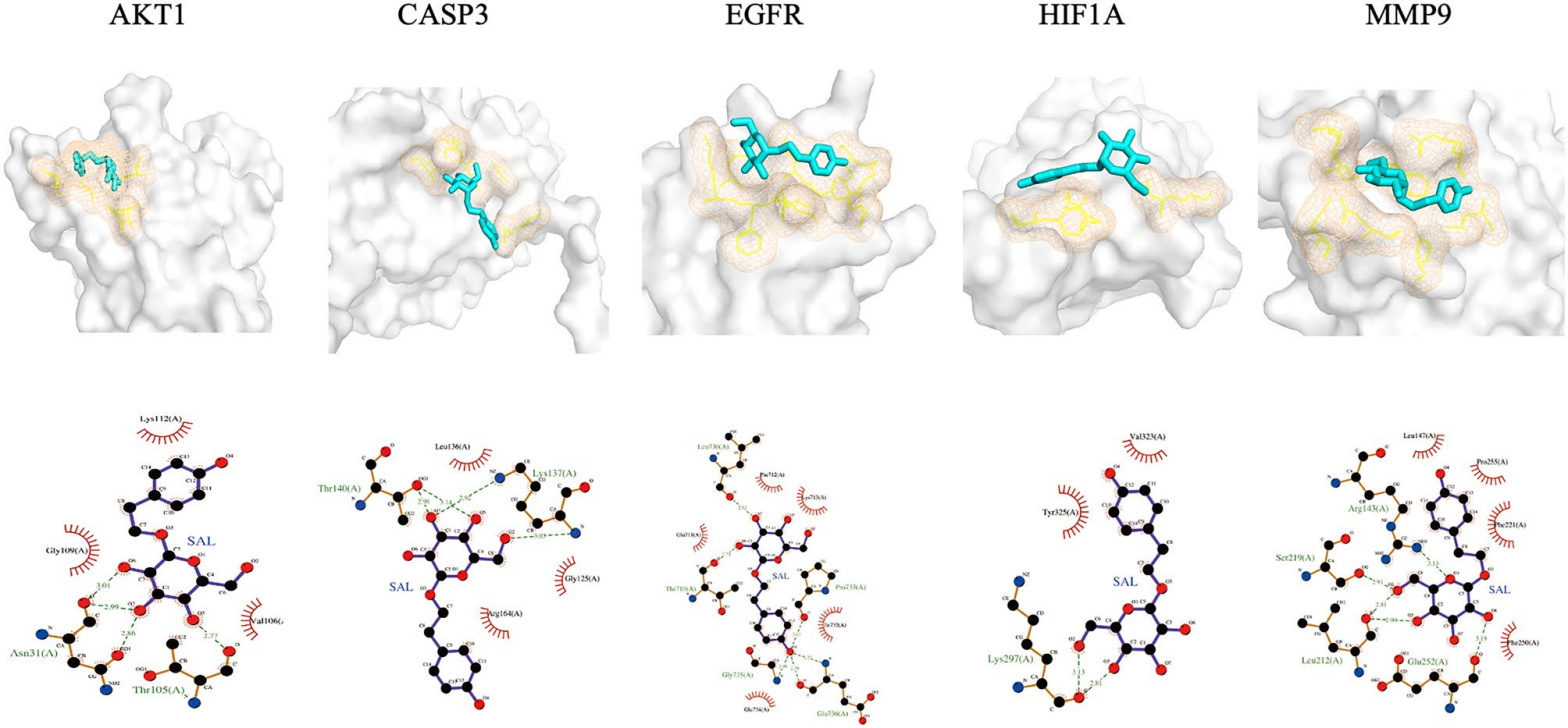
Molecular docking.

We selected the top 5 target genes directly related to the EC for molecular docking to predict the reliability of the interaction between SAL and the target genes. The results of molecular docking showed that SAL could bind stably to EGFR (− 6.1), AKT1 (− 5.6), MMP9 (− 6.4), CASP3 (− 5.9) and HIF1A (− 5.6) (Table 2). The lower the binding energy between small molecule ligands and protein receptors, the better the affinity between them, the more stable. The details of the interaction between drug (ligand) and hub target (receptor) are shown in Fig. 7.

**Table 2:** Molecular docking binding energies.

Gene name	PDB	Vina score	Hydrogen bonding interactions
AKT1	1UNQ	− 5.6	Asn31(A), Thr105(A)
CASP3	2DKO	− 5.9	Thr140(A), Lys137(A)
EGFR	8A27	− 6.1	Leu730(A), Thr710(A), Pro733(A), Gly735(A)
HIF1A	4H6J	− 5.6	Lys297(A)
MMP9	6ESM	− 6.4	Arg143(A), Ser219(A), Leu212(A), Glu252(A)

## Discussion

In recent years, the morbidity and mortality rates of EC have been increasing year by year, seriously affecting the female reproductive health and causing serious social burden. There are no particularly effective treatment options for advanced and recurrent EC. Therefore, the development of navel natural drugs is still needed for the treatment of EC. Studies have shown that traditional Chinese medicine has been widely used to treat various cancers due to its multiple advantages such as low side effects and multiple targets^7,8^. SAL is a natural glycoside compound with anticancer effects, but its application to EC has not been extensively studied. In this article, the pharmacological effects, and potential mechanisms of SAL in the treatment of EC were explored through a network pharmacology combined with molecular docking verification study.

Network pharmacology enables to explore theoretically the mechanism of drug action, and combined with molecular docking research methods can develop new drugs for disease target genes. Using the network pharmacology approach, we obtained 113 potential target genes in SAL for the treatment of EC. The PPI network of 113 intersection genes was further constructed. In addition, we are known to use CytoHubba that AKT1, CASP3, EGFR, HIF1A, and MMP9 may be the potential key target genes of SAL for the therapy of EC.

Serine/threonine kinase (AKT1), also known as protein kinase B, is a key member of the PI3K/AKT signaling pathway. AKT family has three main isoforms: AKT1 (PKBα), AKT2 (PKBβ) and AKT3 (PKBγ), which are closely related to each other and are involved in various cellular processes, such as cell proliferation, apoptosis, cell metabolism and protein transcription, etc.^28^. In addition, AKT1 also plays a central role in tumorigenesis effect. Previous studies have found that AKT is closely associated with tumor progression in ovarian, pancreatic, and gastric cancers^29–31^. In addition, AKT expression level is closely related to pathological grade of malignant tumor, distant metastasis, chemotherapy resistance, progression-free survival, and overall survival rate^32,33^. Recent studies have shown that AKT1 mutations are associated with endometrial dysplasia, leading to benign and early malignant lesions. Therefore, AKT1 is a promising therapeutic target for EC. The PI3K/AKT signaling pathway is a classical pathway that regulates apoptosis in cancer cells by affecting the activity of downstream apoptosis-related molecules (bcl2, bax, caspase3, caspase9). Caspase-3(CASP3) is a key gene involved in the cysteine activation cascade reaction responsible for apoptosis. It is frequently elevated in patients with endometrioid carcinoma, especially in and advanced patients, suggesting its involvement in progressive dysregulation of proliferation and apoptosis, leading to the progression of simple/complex endometrial hyperplasia to cancer^34^. It was shown that in SAL treatment of human hepatocellular carcinoma cell line 97H, SAL decreased the phosphorylation of PI3K and AKT proteins and upregulated the protein expression levels of caspase3 and caspase9^10^. Epidermal growth factor receptor (EGFR) is a common tyrosine kinase receptor that plays an important role in cancer. EGFR and its downstream signaling pathways are involved in the proliferation, migration, and epithelial-mesenchymal transition (EMT) of various cancer cells^35–37^. Studies have shown that EGFR is highly expressed in EC and is closely related to the staging, prognosis, and drug resistance of EC^35,36,38^. Therefore, EGFR is a potential target for the treatment of EC. Hypoxia-inducible factor 1α (HIF1A) is a key transcription factor regulating cellular responses to hypoxia. In general, the expression level of HIF1A in cells is very low, but it is highly expressed in tumor cells and participates in various biological processes of tumor cells^39,40^. The important role of the hypoxic tumor microenvironment in driving tumor progression and the development of therapeutic resistance has recently been recognized and has a key role in the tumor stroma HIF-1α, leading to an increasing clinical research focus on discovering new therapies that inhibit this protein or its targets^41^. Well-known antitumor substances such as LY294002 (a PI3K inhibitor) and tamsulosin (an HSP90 inhibitor) can be used for radiation therapy by downregulating HIF-1α and sensitizing tumors^42,43^. Studies have shown that HIF1A is highly expressed in EC tissues and cell lines, and the high expression of HIF1A is usually closely related to angiogenesis, invasion and prognosis of type I endometrial cancer^44,45^. In endometrial cancer cell lines, inhibition of the PI3K pathway has been shown to sensitize cancer cells to radiotherapy through downregulation of HIF-1α. In addition, direct inhibition of HIF-1α increases cell death after radiotherapy, suggesting that combined inhibition of PI3K- and HIF-1α may enhance the therapeutic effect. Zeng et al. showed that SAL can effectively enhance the growth inhibitory activity of DOX in HeLa-ADR cells by modulating the PI3K/Akt/HIF-1α pathway and the DOX-induced drug resistance pathway^46^. Matrix metallopeptidase 9 (MMP9) is a metalloprotease that plays a key role in various diseases. Studies have shown that MMP9 is involved in various biological processes such as tumor invasion, metastasis, angiogenesis, and tumor microenvironment^47–50^. Furthermore, a meta-analysis showed that MMP9 overexpression may be closely related to the clinical progression and poor prognosis of EC^47^. In SAL-treated human lung cancer cells (A549) and human bladder cancer cells (T24), a significant reduction in MMP-2 and MMP-9 activity was found^15,51^. SAL was also shown to increase tissue inhibitors of metalloproteinase-2 (TIMP-2), a key regulator of MMP-2 and MMP-9 activation^52^. Therefore, SAL may exert anti-tumor effects by inhibiting the metastasis of EC cell lines through MMP9.

To further explore the interactions among target genes involved in SAL anti-EC, we performed KEGG pathway analysis. KEGG analysis showed that the potential pathways for SAL to treat EC are PI3K/AKT signaling pathway, pathway in cancer, apoptosis, MAPK signaling pathway, and FOX signaling pathway. The PI3K-AKT signaling pathway is a key pathway for cancer therapy and is involved in various biological processes such as apoptosis, cell proliferation, and cell cycle^53–55^. Among solid tumors, endometrial cancers had the highest rate of alterations in the PI3K/AKT/mTOR pathway, with 92% of type I and 60% of type II endometrial cancers having specific alterations in this pathway, indicating that PI3K/AKT plays an important role in the pathogenesis of EC^53^. Typically, PI3K/AKT/mTOR pathway overactivation is due to loss of function of upstream tyrosine kinase growth factor receptors, phosphatases and tension homologs (PTEN), PI3KCA amplification or mutation, elevated PIK3R1, AKT gene and mTOR expression^56,57^. PTEN mutations play a key role in the pathogenesis of type I endometrial cancer, while mutations found in mTOR may be mainly involved in the pathogenesis of type II endometrial cancer. Since the PI3K/AKT/mTOR pathway has a high mutation rate in endometrial cancer and all of them are kinases, targeting the three major molecules of this pathway, PI3K/AKT/mTOR, has become a hot research topic for the treatment of endometrial cancer in recent years. Therefore, blocking or inhibiting the PI3K/AKT signaling pathway may be a feasible molecular target for the development of new drugs. We carried out molecular docking on the five key target genes, and the results showed that SAL has good binding force with these target genes, which suggested that SAL may play an anti-tumor effect by inhibiting the core targets and their related signaling pathways.

## Conclusion

Studies have shown that SAL has inhibitory effects on a variety of tumor cells. However, our study is the first to systematically analyze the anti-EC effect and mechanism of SAL through network pharmacology. This study provides a theoretical basis for further experimental studies on the role of SAL in anti-EC, suggesting that SAL is a potential anti-tumor drug in the occurrence and development of EC.

### Supplementary Information

Supplementary Information.

## Data Availability

All data generated or analyzed during this study are included in this article.
